# Is telomere length in peripheral blood lymphocytes correlated with cancer susceptibility or radiosensitivity?

**DOI:** 10.1038/sj.bjc.6604085

**Published:** 2007-11-13

**Authors:** J Barwell, L Pangon, A Georgiou, Z Docherty, I Kesterton, J Ball, R Camplejohn, J Berg, A Aviv, J Gardner, B S Kato, N Carter, D Paximadas, T D Spector, S Hodgson

**Affiliations:** 1 Medical and Molecular Genetics Department, Guy's Hospital, London SE1 9RT, UK; 2 King's College London School of Medicine, Department of Oncology, Rayne Institute, London SE1 7EH, UK; 3 Clinical Genetics Department, Ninewells Hospital Medical School, Dundee DD1 9SY, UK; 4 Hypertension Research Center, University of Medicine and Dentistry of New Jersey, 185 South Orange Ave, Newark, NJ 07103-2714, USA; 5 King's College London, Twin Research & Genetic Epidemiology Unit, St Thomas' Hospital Campus, Lambeth Palace Road, London SE1 7EH, UK; 6 Clinical Genetics, St George's University of London, Cranmer Terrace, London SW17 0RE, UK

**Keywords:** telomere, breast cancer susceptibility, radiosensitivity, chromosome, apoptosis

## Abstract

Mean terminal restriction fragment (TRF) lengths in white blood cells (WBCs) have been previously found to be associated with breast cancer. To assess whether this marker could be used as a test for breast cancer susceptibility in women, TRF length was measured in 72 treated female breast cancer patients and 1696 unaffected female controls between the ages of 45 and 77 from the Twin Research Unit at St Thomas' Hospital, as well as 140 newly diagnosed breast cancer cases and 108 mammographically screened unaffected controls from Guy's Hospital. Mean TRF was also tested for correlation with chromosome radiosensitivity and apoptotic response in the Guy's Hospital patients. After adjusting for age, smoking and body mass index, there was no significant difference in TRF lengths between the treated breast cancer patients and unaffected controls (*P*=0.71). A positive correlation between age-adjusted apoptotic response and mean TRF in newly diagnosed untreated breast cancer patients (*P*=0.008) was identified but no significant difference in TRF lengths between breast cancer patients and unaffected controls was detected (*P*=0.53). This suggests that TRF lengths in WBC, is not a marker of breast cancer susceptibility and does not vary significantly between affected women before and after treatment.

Telomeres, a series of TTAGGG repeat sequences, are located at the end of chromosomes and prevent these being recognised as double-strand breaks (Blackburn, 2001). We have previously shown that telomere length in white blood cells (WBC) is inversely correlated with age, smoking and body mass index (BMI) (Benetos *et al*, 2001; Valdes *et al*, 2005). A link between mean terminal restriction fragment (TRF) lengths in WBC and cancer susceptibility is, however, less certain (Wu *et al*, 2003). DNA damage response proteins are thought to be involved in telomere maintenance (Slijepcevic, 2004) and this suggests that telomere shortening could be related to breast cancer susceptibility. It is possible that short telomeres reflect increased cell turnover or the loss of key/critical genes in the telomeric region, which could lead to a failure of apoptosis or the development of chromosomal instability (Kim *et al*, 2002; Preto *et al*, 2004). Meeker and Argani (2004) demonstrated that somatic telomere length is significantly shorter in premalignant ductal carcinoma *in situ* than in normal breast tissue.

An association of short telomeres with hypersensitivity to ionising irradiation has been reported previously (Goytisolo *et al*, 2000; Bouffler *et al*, 2001) and it has been suggested that it could be important in determining tissue sensitivity to radiotherapy (Wu *et al*, 2003). Telomere length in radiosensitive murine lymphoma cells L5178Y-S has been shown to be seven-fold shorter than in radioresistant cells and a significant inverse correlation between telomere length and chromosomal radiosensitivity has previously been observed in lymphocytes from 24 breast cancer patients and 5 normal individuals (McIlrath *et al*, 2001).

This link between radiosensitivity and telomere shortening in peripheral blood lymphocytes could be due to a defect in homologous recombination repair of both double-strand breaks and telomere uncapping (Wong *et al*, 2000).

To determine whether mean TRF is associated with breast cancer susceptibility, we assessed mean TRF in WBCs from 72 treated (female) breast cancer patients and 1696 unaffected female controls between the ages of 45 and 77 from the Twin Research Unit at St Thomas' Hospital. To exclude the possible effect of chemotherapy/radiotherapy or surgery on these assays mean TRF was also measured in 140 newly diagnosed untreated breast cancer patients and 108 age, sex and ethnically matched controls.

We also present the correlation between TRF and two *in vitro* markers of radiosensitivity (chromosome radiosensitivity and induced apoptotic response to irradiation) in peripheral blood lymphocytes from these newly diagnosed breast cancer patients and unaffected controls, and discuss their significance.

## MATERIALS AND METHODS

### Recruitment of breast cancer treated individuals and unaffected controls

Data were available on 7674 female twins who reported if they had ever been diagnosed with breast cancer. These data came from four sources, namely twin interviews, questionnaire 2 (1999), questionnaire 7 (2002) and baseline health questionnaire from Twins UK (2004–present). Of the 7674 twins, 2597 had measurements on TRF length and therefore the rest of the report will deal with these 2597 subjects (72 had been treated previously for breast cancer, 2525 had never had breast cancer).

### Recruitment of newly diagnosed breast cancer individuals and unaffected controls

Terminal restriction fragment lengths were measured in DNA extracted from the WBCs of 140 newly diagnosed untreated female breast cancer patients (mean age=59, range=27–85) with no previous history of cancer and before treatment with radiotherapy or chemotherapy and 108 age- and ethnicity-matched female controls with a normal mammogram in the last 6 months. The unaffected controls (mean age=55, range=26–83) were recruited as close as possible in time to the cases, from breast outpatient and mammographic screening clinics.

### Mean TRF measurement

The mean TRF lengths were then age-adjusted and correlated with two *in vitro* markers of radiosensitivity, apoptotic response to irradiation and chromosome breakage using the G2 assay. Mean TRF length, radiosensitivity, apoptotic response to irradiation and chromosome breakage were available in 123 newly diagnosed breast cancer patients and 108 unaffected controls. The coefficient of variation of the TRF length measurement assay in this study was 1.4%.

The methods and results of testing for chromosome radiosensitivity and apoptotic response to irradiation in these newly diagnosed breast cancer patients and controls are described in more detail in Docherty *et al* (2007). They are described in brief as follows:

### G2 radiosensitivity assay

This was carried out according to Howell and Taylor (1992). Six whole blood cultures from heparinised blood samples were incubated for 65 h at 37°C. The cells were irradiated using a caesium-137 source at 0.5/1 Gy, or mock treated. The cells were re-incubated for 30 min or 3 h. Colcemid was added and harvested 1 h later using KCl and methanol/acetic acid fixative. The slides were stained with leishmans and 50 metaphases were scored blind for chromatid breaks.

### Apoptotic assay

Peripheral blood lymyphocytes were separated by centrifugation on histopaque and cultured for 70 h. The cells were irradiated with 4 Gy using a caesium-137 source or mock treated. The cells were re-cultured for a further 24 h and then fixed in 70% ethanol. The ethanol was removed and the DNA was denatured in HCl and stained for DNA content with propidium iodide. DNA content was assessed by flow cytometry.

### Statistical analysis

Standard multiple regression techniques were used to investigate associations between TRF length and age- and age-adjusted TRF length with apoptotic response and disease status. Stata 9.0 software was used. The regression cluster option in Stata was used to account for correlation within twin pairs. Apoptotic response to irradiation and age-adjusted mean TRFs were correlated using linear regression analysis. With 108 breast cancer cases and 140 controls, a difference of 200 bp would be detected with a power of 62% at a 0.05 level of significance.

Out of the 1768 individuals, 1713 had two TRF measurements taken. A paired *t*-test comparing intra-individual TRF measurements using the Southern blot technique from the Twins Unit treated breast cancer patients and unaffected controls was carried out. On average there was an extremely small (0.009 kb) but significant difference between the sample measurements from the same individuals (*P*=0.0322). This is very unlikely to be the cause of a type I error in this study and a true significant difference between cases and controls missed.

## RESULTS

### TRF in breast cancer patients post-treatment and unaffected controls

Complete data on TRF length were available for 2525 controls and 72 breast cancer treated patients. However, the mean age of the cases was significantly greater than that of controls (56.3 *vs* 47.9, *P*<0.001). To obtain a better age match for the cases and controls, we considered only controls aged between 45 and 77 years. This rendered a sample of 1768 (1696 controls and 72 cases) subjects with no significant difference between the mean ages in each group (56.27 *vs* 55.09, *P*=0.22).

**Figure 1:**
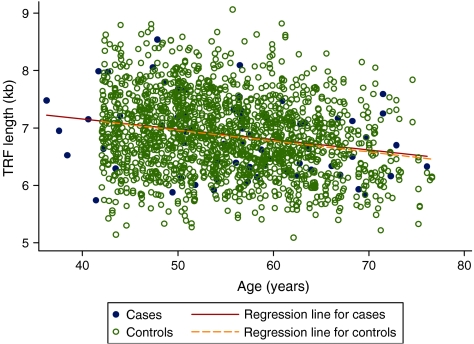
Relationship between mean terminal restriction fragment (TRF) length in white blood cell (WBC) and age in 72 women post-breast cancer treatment and 1696 healthy controls recruited from the Twins Unit at St Thomas' Hospital.

The relationship between TRF length and age in all subjects is shown in Figure 1. Overall TRF length decreased with age in both groups with a correlation in cases of −0.27 and controls −0.22. After adjusting for age, there was no significant difference in mean TRF lengths of subjects who had had breast cancer and those who had never had breast cancer (6.859 kb *vs* 6.858, *P*=0.988).

### TRF in newly diagnosed breast cancer patients and unaffected controls

Terminal restriction fragment length was successfully measured in 248 subjects, 140 newly diagnosed, untreated female breast cancer patients and 108 age, sex and ethnically matched controls. All the subjects were female and cases were on average older than controls (57.3 *vs* 54.0 years, *P*=0.034).

**Figure 2:**
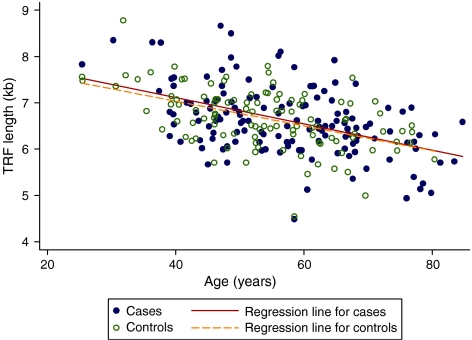
Relationship between mean terminal restriction fragment (TRF) length in white blood cell (WBC) and age in 140 newly diagnosed and untreated breast cancer cases and 108 unaffected controls with a normal mammogram in the previous 6 months.

The relationship between TRF length and age in these subjects is shown in Figure 2. Overall TRF length decreased similarly with age in both groups with a correlation in cases of −0.47 and controls −0.49. A comparison of average TRF lengths of cases and controls adjusting for age revealed that the age-adjusted TRF length of breast cancer cases and controls did not differ (6.65 kb for cases *vs* 6.60 for controls; *P*=0.529).

### Correlation between mean TRF length and chromosome radiosensitivity and apoptotic response to irradiation

There was no significant correlation between radiosensitivity and TRF length in 123 newly diagnosed, untreated breast cancer patients and 102 unaffected controls.

**Figure 3:**
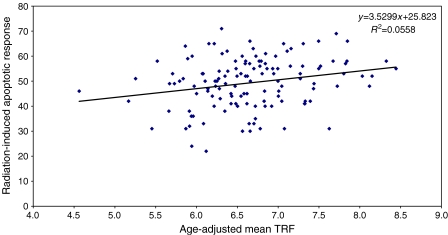
Correlation of percentage peripheral blood lymphocyte apoptotic response to irradiation to mean age-adjusted white blood cell (WBC) terminal restriction fragment (TRF) length in newly diagnosed breast cancer patients (*N*=123, *r*=0.236, *P*=0.008).

**Figure 4:**
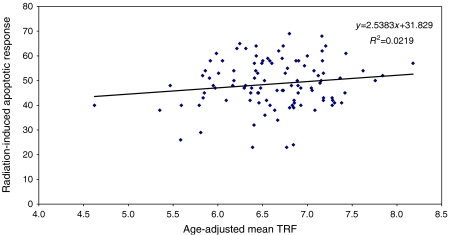
Correlation of percentage peripheral blood lymphocyte apoptotic response to irradiation to mean age-adjusted white blood cell (WBC) terminal restriction fragment (TRF) length in unaffected controls with a normal mammogram in the previous 6 months (*N*=102, *r*=0.148, *P*=0.137).

There was a significant positive correlation between age-adjusted apoptotic response to irradiation and mean telomere length in 123 newly diagnosed breast cancer patients (*r*=0.236, *P*=0.008) (Figure 3). No significant correlation was present between these parameters in 102 unaffected controls (*r*=0.148, *P*=0.137) (Figure 4).

## DISCUSSION

This is the largest study of mean TRF measurement in WBC from breast cancer patients and unaffected controls. It has been demonstrated that loss of telomeres increases chromosomal instability resulting in chromosomal rearrangements associated with carcinogenesis (Murnane, 2006) and tumour cell progression (Lo *et al*, 2002). As a result, telomere length has been used as a marker of disease progression in solid tumours (Bisoffi *et al*, 2006; Fordyce *et al*, 2006). Mean TRF length has previously been shown to decrease with age. However, as previously demonstrated in mouse cell lines, no statistical difference in age-adjusted mean TRF was demonstrated between any of the breast cancer patient groups and unaffected control groups. Therefore, we found no evidence that a reduction or increase in TRF in WBCs is linked to breast cancer susceptibility, as was previously suggested in a smaller study (McIlrath *et al*, 2001).

When the results are controlled for BMI and smoking status, there is no evidence that average TRF length differs between breast cancer cases and controls (*P*=0.707) (Table 1). No association between chromosome radiosensitivity and BMI or smoking was found in the newly diagnosed breast cancer patients and age-matched controls (Docherty *et al*, 2007).

### Why was no difference found between breast cancer cases and controls, as previously suggested?

This is a much larger cohort of age-matched breast cancer patients than previous studies. Pre- and post-treatment breast cancer patients were analysed and compared with controls separately and therefore it is unlikely that treatment itself is the reason why no difference was found.

Our studies used peripheral blood lymphocytes rather than somatic tumour tissue or established cell lines, which may have different TRF measurements and radiosensitivity, respectively. The newly diagnosed breast cancer patients were controlled with patients who had had a normal mammogram reported after a technical repeat mammogram in the previous 6 months. This may give different results compared to other control groups.

We have previously shown that there was no significant difference between apoptotic response to irradiation and chromosome radiosensitivity between newly diagnosed breast cancer cases and controls (Camplejohn *et al*, 1995, 2003). However, in line with these studies, we detected a decline in final induced apoptotic response with age in both patients and unaffected controls (Docherty *et al*, 2007). By combining our results, we have shown a significant positive correlation between age-adjusted apoptotic response to irradiation and mean telomere length in WBC from newly diagnosed breast cancer patients (*P*=0.008). This trend was also observed in the unaffected controls. Loss of telomeric sequences could theoretically alter the cell's ability to undergo apoptosis after irradiation, due to loss of key apoptotic or DNA damage-sensing genes.

Our results demonstrated that the mean TRF and apoptotic response to irradiation in WBC reduce with age, and that age-adjusted mean TRF is correlated with apoptotic response to irradiation. This could be due to shared genetic mechanisms for TRF maintenance and apoptotic response to irradiation. However, within the power constraints of our study, age-adjusted mean TRF in WBCs is not a marker of breast cancer susceptibility.

## Figures and Tables

**Table 1:** Multiple regression analysis with TRF as the dependent variable in breast cancer cases and controls

**Covariate**	**Regression** c**oefficient**	***P*-value**
DS	−0.023	0.776
Smoke	−0.023	<0.0001
BMI	−0.014	0.264

BMI=body mass index; DS=disease status.

DS is coded as 0 and 1 for controls and cases, respectively.

Smoke is coded 0 and 1 for non-smokers and smokers, respectively.
